# Asymptomatic Pulmonary Vein Stenosis: Hemodynamic Adaptation and Successful Ablation

**DOI:** 10.1155/2016/4979182

**Published:** 2016-12-25

**Authors:** John J. Lee, Denis Weinberg, Rishi Anand

**Affiliations:** ^1^University of Miami at Holy Cross Hospital, Fort Lauderdale, FL, USA; ^2^South Florida Multispecialty Associates, LLC, Miami Beach, FL, USA; ^3^Holy Cross Hospital, Fort Lauderdale, FL, USA

## Abstract

Pulmonary vein stenosis is a well-established possible complication following an atrial fibrillation ablation of pulmonary veins. Symptoms of pulmonary vein stenosis range from asymptomatic to severe exertional dyspnea. The number of asymptomatic patients with pulmonary vein stenosis is greater than originally estimated; moreover, only about 22% of severe pulmonary vein stenosis requires intervention. We present a patient with severe postatrial fibrillation (AF) ablation pulmonary vein (PV) stenosis, which was seen on multiple imaging modalities including cardiac computed tomography (CT) angiogram, lung perfusion scan, and pulmonary angiogram. This patient did not have any pulmonary symptoms. Hemodynamic changes within a stenosed pulmonary vein might not reflect the clinical severity of the obstruction if redistribution of pulmonary artery flow occurs. Our patient had an abnormal lung perfusion and ventilation (V/Q) scan, suggesting pulmonary artery blood flow redistribution. The patient ultimately underwent safe repeat atrial fibrillation ablation with successful elimination of arrhythmia.

## 1. Introduction

Pulmonary vein stenosis, which occurs in 1% to 3% of cases, is a well-recognized potential complication after radiofrequency (RF) ablation of pulmonary veins. The clinical manifestations of pulmonary vein stenosis vary widely from asymptomatic to severe exertional dyspnea. While these symptoms are primarily affected by the severity and number of affected veins, pulmonary blood flow redistribution is thought to be one of the factors contributing to blunting clinical symptoms after RF ablation of atrial fibrillation (AF) [1]. We report a case of compensatory hemodynamic change that leads to asymptomatic pulmonary vein (PV) stenosis with subsequent successful ablation.

## 2. Case Report

**Figure 1:**
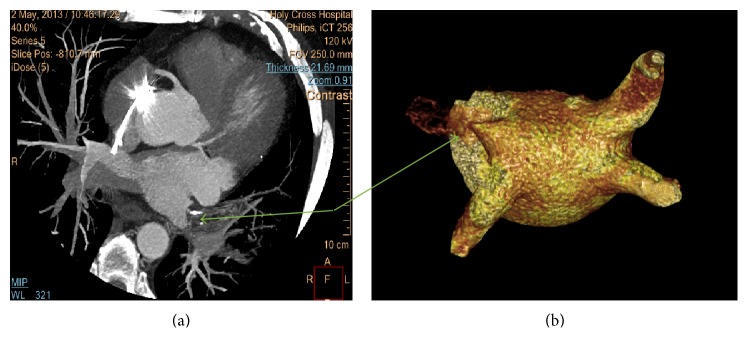
(a) Cardiac c*omputed tomography angiogram.* (b)* 3D reconstruction of the left atrium*. Green arrows indicating filling and perfusion defect of the left superior pulmonary vein suggesting an ostial stenosis.

**Figure 2:**
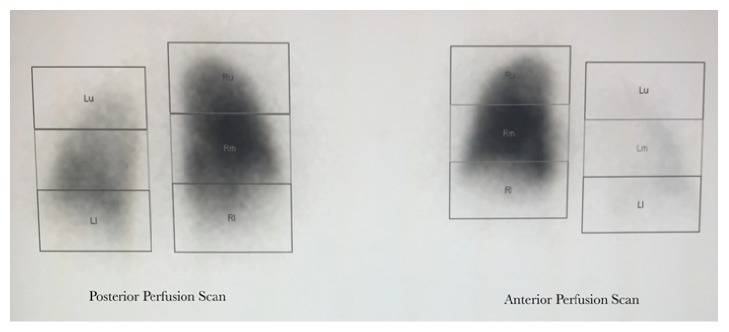
*Lung perfusion scan*. Patient's left lung perfusion was significantly diminished compared to right. This finding suggests development of pulmonary artery blood flow redistribution via systemic arterial collaterals in response to the left superior pulmonary vein stenosis.

**Figure 3:**
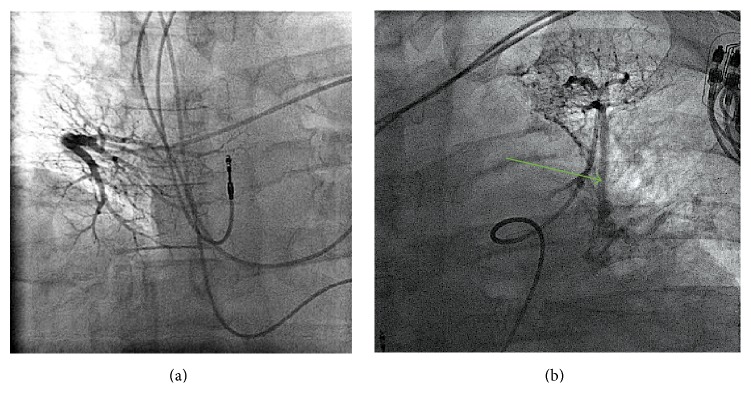
(a)* Normal right pulmonary angiogram*. Venous return confined to the area of the lung supplied by the arterial vasculature. No evidence of collateral development. (b)* Left pulmonary angiogram*. Green arrow demonstrating pulmonary arterial collateral from left upper lobe to the mid segment of the left lung.

A 56-year-old male with history of AF, status after ablation, eight years ago at an outside hospital, presented with recurrent AF. Five years after his initial ablation, the patient developed symptomatic palpitations due to atrial fibrillation and he was prescribed flecainide and metoprolol. Despite antiarrhythmic therapy, the patient continued to have symptomatic atrial fibrillation with palpitations but no shortness of breath. The patient was taken for AF ablation. The preablation echocardiogram demonstrated normal ventricular function and pulmonary pressures. The preprocedural computed tomography (CT) scan along with the three-dimensional (3D) reconstruction was done on the procedural table prior to the transeptal puncture but did not pick up the pulmonary vein stenosis. Pulmonary vein potential mapping noted that there was a potential at the ostium of the left superior pulmonary vein. There was also difficulty advancing the catheter into the left superior pulmonary vein due to a possible obstruction. Direct angiography of the left superior pulmonary vein (LSPV) confirmed complete PV stenosis. The procedure was aborted in lieu of further diagnostic work up. A higher resolution cardiac CT angiogram (Figure 1(a)) and left atrium 3D reconstruction (Figure 1(b)) was used to confirm subtotal occlusion of the left superior pulmonary venous trunk. A lung perfusion scan revealed significantly decreased left upper lung perfusion (Figure 2) with the left lung contributing approximately 18% of the lung function and the right lung contributing 82% of the total lung function. A levophase angiogram of the right middle lobe pulmonary arterial system demonstrated venous return confined to the area of the lung supplied by the arterial vasculature. The levophase pulmonary angiogram demonstrated well developed collateral circulation from the left upper lobe to the mid segment of the left lung (Figures 3(a) and 3(b), resp.). After cardiac workup, it was concluded that this pulmonary vein stenosis is chronically totally occluded and less amenable to percutaneous intervention for reestablishment of flow. Ultimately, the decision to intervene to relieve the stenosis and to repeat AF ablation on the remaining pulmonary veins was left to the patient. The patient initially opted for continuance of antiarrhythmic drug therapy. However, due to persistence of breakthrough atrial fibrillation despite antiarrhythmic therapy, the patient ultimately underwent repeat atrial fibrillation ablation procedure two years after his initial presentation to our institution.

## 3. Discussion

The frequency of PV stenosis, a well-established possible complication following an AF ablation of pulmonary veins, has been declining due to the improvement of technique. However, depending on the technique and diagnostic modalities used, PV stenosis occurs as often as 40% of patients who underwent AF ablation [1]. PV stenosis acquired after AF ablation varies in severity from asymptomatic to nonspecific symptoms including persistent cough, hemoptysis, and exertional dyspnea [2]. Given these nonspecific clinical symptoms, physicians should be highly suspicious of the diagnosis of PV stenosis in postablation patients and further evaluate patients with multiple imaging modalities. In most cases of PV stenosis, including severe cases, clinical symptoms improve without intervention presumably due to the involvement of pulmonary artery blood flow redistribution [1]. Only about 22% of severe PV stenosis, defined as more than 50% luminal occlusion, requires intervention [3]. Most patients improve spontaneously likely due to the compensatory hemodynamics [1]. Although it is hard to demonstrate PV stenosis and its hemodynamic adaptation by imaging, the absence of perfusion on a lung perfusion scan can be suggestive of pulmonary artery to systemic collaterals (Figure 2) [4, 5]. Such hemodynamic changes are thought to be similar to those adaptations observed in congenital PV atresia [6]. In congenital unilateral PV atresia, due to increased venous pressure, there is retrograde flow of the ipsilateral pulmonary artery creating systemic to pulmonary arterial collaterals [6]. This would make the perfusion of the affected lung absent on a perfusion scan, as it was observed in our patient's perfusion scan [6]. Moreover, we suggest that the collateral vasculature (Figure 3(b)), connecting the left upper pulmonary artery to the left lower pulmonary artery, is for lung parenchymal preservation.

**Figure 4:**
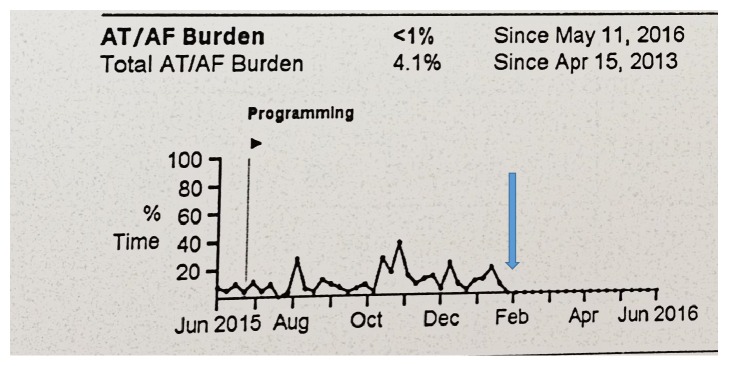
*Blue arrow *indicates repeat atrial fibrillation ablation in January 2016. Note that arrhythmia logbook from patient's pacemaker demonstrates no further recurrence of atrial fibrillation nearly six months after procedure.

Ultimately, the patient underwent successful repeat atrial fibrillation ablation employing a wide antral circumferential ablation technique with successful resolution of the left superior pulmonary vein. The patient's pacemaker was interrogated six months after ablation and demonstrated successful elimination of atrial fibrillation (Figure 4).

## 4. Conclusion

We present a patient with severe post-AF ablation PV stenosis without pulmonary symptoms.

Our patient's perfusion scan, which demonstrated no perfusion of the affected lung, is suggestive of pulmonary artery blood flow redistribution via pulmonary artery to systemic collaterals. The patient ultimately underwent safe repeat atrial fibrillation ablation with successful elimination of arrhythmia.
